# Progress and future of in vitro models to study translocation of nanoparticles

**DOI:** 10.1007/s00204-015-1518-5

**Published:** 2015-05-15

**Authors:** Hedwig M. Braakhuis, Samantha K. Kloet, Sanja Kezic, Frieke Kuper, Margriet V. D. Z. Park, Susann Bellmann, Meike van der Zande, Séverine Le Gac, Petra Krystek, Ruud J. B. Peters, Ivonne M. C. M. Rietjens, Hans Bouwmeester

**Affiliations:** Department of Toxicogenomics, Maastricht University, PO Box 616, 6200 MD Maastricht, The Netherlands; Centre for Health Protection, National Institute for Public Health and the Environment (RIVM), PO Box 1, 3720 BA Bilthoven, The Netherlands; Division of Toxicology, Wageningen University, Tuinlaan 5, 6703 HE Wageningen, The Netherlands; AMC, Coronel Institute of Occupational Health, Academic Medical Centre, University of Amsterdam, 1105 AZ Amsterdam, The Netherlands; TNO, Utrechtseweg 48, 3704 HE Zeist, The Netherlands; RIKILT- Wageningen UR, PO Box 230, 6700 AE Wageningen, The Netherlands; UT BIOS, Lab on a Chip Group, MESA+ Institute for Nanotechnology, MIRA Institute for Biomedical Engineering and Technical Medicine, University of Twente, Enschede, The Netherlands; Philips Innovation Services, High Tech Campus 11, 5656 AE Eindhoven, The Netherlands

**Keywords:** In vitro models, Nanoparticles, Toxicokinetics, Lung, Oral, Dermal, Placenta

## Abstract

The increasing use of nanoparticles in products likely results in increased exposure of both workers and consumers. Because of their small size, there are concerns that nanoparticles unintentionally cross the barriers of the human body. Several in vivo rodent studies show that, dependent on the exposure route, time, and concentration, and their characteristics, nanoparticles can cross the lung, gut, skin, and placental barrier. This review aims to evaluate the performance of in vitro models that mimic the barriers of the human body, with a focus on the lung, gut, skin, and placental barrier. For these barriers, in vitro models of varying complexity are available, ranging from single-cell-type monolayer to multi-cell (3D) models. Only a few studies are available that allow comparison of the in vitro translocation to in vivo data. This situation could change since the availability of analytical detection techniques is no longer a limiting factor for this comparison. We conclude that to further develop in vitro models to be used in risk assessment, the current strategy to improve the models to more closely mimic the human situation by using co-cultures of different cell types and microfluidic approaches to better control the tissue microenvironments are essential. At the current state of the art, the in vitro models do not yet allow prediction of absolute transfer rates but they do support the definition of relative transfer rates and can thus help to reduce animal testing by setting priorities for subsequent in vivo testing.

## General introduction

Nanoparticles have attractive and novel properties compared with their bulk counterparts and are therefore used in an increasing number of consumer products (Nanotechnologies 2014). Examples are zinc oxide and titanium dioxide nanoparticles in sunscreens and silver nanoparticles in food packaging material, textiles, and cosmetics, but many more have been identified (Bouwmeester et al. 2014). The increasing use of nanoparticles in products likely results in increasing exposure of both workers and consumers. Because of the unique properties of nanoparticles that are related to their small size, concerns arise that nanoparticles would unintentionally cross the barriers of the human body, which would result in internal exposure to nanoparticles potentially leading to adverse effects.

Several in vivo studies have been performed to assess the distribution of nanoparticles after inhalation, oral exposure, skin exposure, and intravenous injection (Balasubramanian et al. 2010; Braakhuis et al. 2014a; Creutzenberg et al. 2012; De Jong et al. 2008; Elder et al. 2006; Geraets et al. 2012; Kreyling et al. 2009; Leite-Silva et al. 2013; Ma-Hock et al. 2012; Oberdorster et al. 2004; Semmler et al. 2004; Takenaka et al. 2001; van der Zande et al. 2012, 2014). These studies show that, depending on the exposure route, time, concentration, as well as on their characteristics, nanoparticles can cross the lung, gut, skin, and placental barrier.

Information on the kinetics of nanoparticles in the human body is essential for risk assessment purposes, because of their potency to accumulate. The overall resultant of absorption, distribution, metabolism, and excretion (ADME), i.e. internal exposure, will determine target tissue doses and will be critical for the ultimate systemic adverse health effects (Geraets et al. 2014). Even in cases of low absorption of nanoparticles, the often chronic nature of the exposure (inhalatory, oral or dermal) might result in internal accumulation of the nanoparticles potentially reaching levels that might give rise to health concerns (van Kesteren et al. 2014). The current risk assessment of nanoparticles (and chemicals) mainly relies on in vivo studies using animal models (EFSA 2011). While these in vivo studies provide unique information on the distribution of nanoparticles in a whole organism, the number of animal studies should be reduced as much as possible for several reasons (Hartung et al. 2013). First, the use of animals is ethically debatable. Secondly, animal models do not fully simulate the physiology of humans. Lastly, given the great number of and variety in different nanoparticles, it is impossible and economically not feasible to test all of them through in vivo studies. Therefore, in vitro models have been developed to study the translocation of nanoparticles (Hartung et al. 2013) and estimate the in vivo internal exposure. However, before such in vitro models can reliably be used in risk assessment of nanoparticles, they need to be well described and validated (Kandarova and Letasiova 2011; Worth and Balls 2004) using in vivo data (Genschow et al. 2002).

To obtain reliable NP kinetic data from in vitro or in vivo studies, robust analytical detection methods should be used in the experiments. Over the last couple of years, the quality of NP characterization methods used in kinetic studies has been improved, but much is to be gained here. Therefore, we reviewed the current state of the knowledge on analytical detection methods and proposed directions for further improvement and incorporation in in vitro or in vivo studies.

In this review we aimed to evaluate the existence and performance of in vitro models that mimic the barriers of the human body. Where possible we compare the observed translocation in vitro to the in vivo translocation to compare to what extent the in vitro results mimic the in vivo situation. In addition, we have included the placental barrier that protects the unborn foetus from exposure via the maternal circulation. For an overview on the status of alternatives for regulatory toxicology in general, we refer to the 2014 JRC Science and Policy Report by Worth et al. (2014). We first describe the different in vitro models that are currently in use to study the transfer of nanoparticles via inhalation, oral uptake, skin uptake, and placental uptake. In vitro models that are used only to assess the toxicity of nanoparticles, but not to measure translocation, are excluded from this review. After defining the in vitro models available to study transfer across the different barriers, we compare the results of the in vitro models with available in vivo data and discuss their predictive value. Finally, we give recommendations for the future development of relevant in vitro models.

## Introduction to the lung barrier

The main function of the lungs is to transport oxygen from the atmosphere into the bloodstream and to release carbon dioxide from the bloodstream into the atmosphere. During inhalation, air travels from the mouth or nose through the nasopharynx, oropharynx, larynx, and trachea. The trachea divides into two main bronchi, which branch to the left and right lungs and subsequently subdivide into a system of bronchi and bronchioles until the alveoli where the gas exchange takes place. The airways are lined by ciliated respiratory epithelium, which is covered by a mucus layer. The mucociliary movement is an important clearance mechanism, especially to remove inhaled (nano)particles. Deeper in the airways, the clearance is slower, given the increased pathway length and decreased mucous velocity (Geiser and Kreyling 2010). Nanoparticles, especially those that dissolve readily such as ZnO, may be able to translocate the mucus layer and reach the epithelial cells and thus cause local damage (Frieke Kuper et al. 2015; Landsiedel et al. 2014a; Vandebriel and De Jong 2012). They may also be able to cross the epithelial barrier and reach underlying interstitium with its blood and lymph vasculature.

The alveoli are lined by a single epithelial layer under which is an interstitium with extracellular matrix, blood capillaries, and stromal cells. The epithelial layer is covered by surfactant at the alveolar luminal side. Alveolar type I cells form the structure of the alveolar wall. These cells are very thin to improve the gas exchange. In addition, type I cells have tight junctions to prevent chemicals and particles from entering the bloodstream. Besides type I cells, alveolar type II cells secrete pulmonary surfactant to lower the surface tension. Finally, for clearance of particles and pathogens from the lungs, alveolar macrophages are present (Klein et al. 2011; Möller et al. 2010).

To cross the lung–blood barrier, nanoparticles must deposit in the alveolar region. The deposition of inhaled particles depends on the morphology of the lungs, the respiratory conditions, and the physicochemical properties of the particles. The most important physicochemical properties of inhaled particles that influence deposition are (agglomerate) size, size distribution, density, shape, charge, and hygroscopicity (Braakhuis et al. 2014b; Carvalho et al. 2011; Pilcer and Amighi 2010). When the agglomerate size of nanoparticles is <100 nm but >10 nm, a considerable part will deposit in the alveolar region (about 30 % of the particles) (Asgharian et al. 2009; ICRP 1994; Oberdorster 1989). Below 30 nm, the deposition shifts from the alveoli more towards to tracheobronchial region (Braakhuis et al. 2014b).

Once deposited in the alveoli, nanoparticles can be cleared from the lungs by alveolar macrophages. However, single nanoparticles and agglomerates of <100 nm are less efficiently phagocytized by alveolar macrophages compared with microparticles or large agglomerates of >1 µm (Bakand et al. 2012; Muhlfeld et al. 2008; Phalen et al. 2010). After uptake of the particles, macrophages can move gradually upward by the mucociliary escalator, are subsequently swallowed, and enter the gastrointestinal tract. If not cleared by phagocytosis, nanoparticles can be taken up by the alveolar epithelium and reach the pulmonary interstitium from which they are transported to the local lymph nodes, or reach the blood circulation (Borm et al. 2006). Translocated particles may subsequently reach organs where they can be taken up and might cause damage (Braakhuis et al. 2014b).

## Introduction to the intestinal barrier

The primary functions of the human gastrointestinal (GI) tract are related to the digestion and absorption of nutrients and electrolytes, and to water homeostasis. The GI tract is responsive to internal stimuli as well as to (microbe) stimuli from the lumen content. The GI epithelial layer forms a tight, but selective barrier: nutrients are absorbed efficiently, while microbes, for example, are not. Anatomically the gut wall can be divided into the mucosa, submucosa, muscularis externa, and serosa. At the lumen site, the gut wall consists of a mucosa, which is a combined mucus and cell epithelial layer. The composition of the mucus and the type of cells is variable along the GI tract and reflects the specialized function of each region. The submucosa is a layer of connective tissue that contains lymphatic and blood vessels as well as ganglion and nerve cells. In the next layer, the muscularis externa, the main smooth muscles of the gut are found. The thickness of the muscle layer varies. The serosa is a squamous epithelium (mesothelium) which sits on connective tissue and is continuous with the abdominal peritoneum.

The small intestine is the site where most of the chemical and mechanical digestion takes place and where almost all of the absorption of nutrients and electrolytes is carried out. The wall of the small intestine is lined with absorptive mucosa. The mucosal surface is extended by the presence of crypts and villi. The most common epithelial cell is the enterocyte, its major function being to absorb nutrients. The second cell type is the mucus-secreting goblet cell, the mucus acts as a lubricant and protects the mucosa from irritation. Lastly, the gut-associated lymphoid tissue (GALT) includes several specialized cells including Peyer’s patches, M cells, and intraepithelial lymphocytes, which are part of the intestinal immune system.

Following ingestion, translocation of particles into and across the gastrointestinal mucosa can occur via four different: (1) via endocytosis, through enterocytes, (2) via the M cell-rich layer of Peyer’s patches (small intestinal lymphoid aggregates), (3) via persorption, where particles can translocate through a ‘hole’ left in the epithelium when enterocytes shed from the villous tip, and (4) via the paracellular route, where nanoparticles pass across tight junctions of the epithelial cell layer (Powell et al. 2010). While the exposure of the gastrointestinal mucosa to engineered nanoparticles might pose yet unresolved heath issues, it is important to realize that people in the Western world are daily exposed to sub-micrometre-sized mineral particles (Powell et al. 1996, 2010). These particles have been observed to be composed of aluminosilicates, titanium dioxide, and a small percentage of non-aluminium-containing silicates such as silica (Si0_2_) and magnesium trisilicate (talc) (Dekkers et al. 2011; Powell et al. 1996).

## Introduction to the skin barrier

**Fig. 1:**
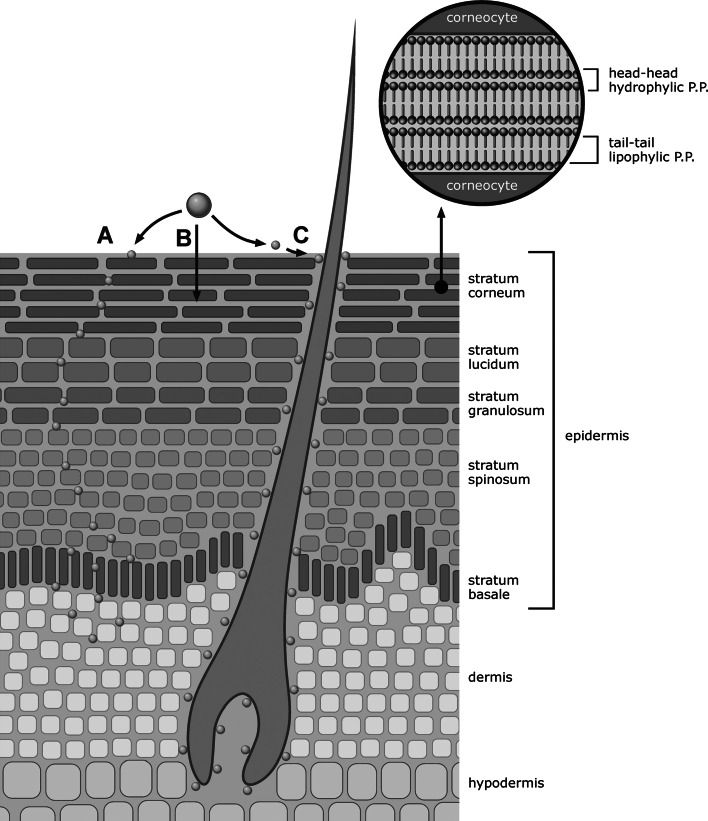
*A* Schematic illustration of the skin and main penetration routes, insert showing the lipid bilayers between corneocytes. Route *A*: across the lipid bilayers (intercellular route); Route *B*: across the corneocytes and lipid bilayers (intracellular route); Route *C*: along hair follicles and sweat glands

**Fig. 2:**
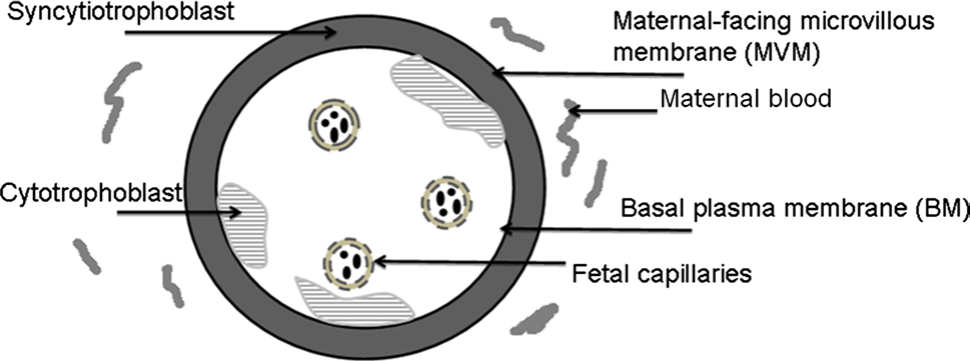
Schematic illustration of the placental barrier as a cross section of a human placental villus. The placental barrier consists of two layers: the syncytiotrophoblast and cytotrophoblast, the latter forming a discontinuous layer. The basal plasma membrane (BM) of the syncytiotrophoblast is oriented towards the foetal circulation, while the maternal-facing microvillous plasma membrane (MVM) faces the maternal blood compartment

**Fig. 3:**
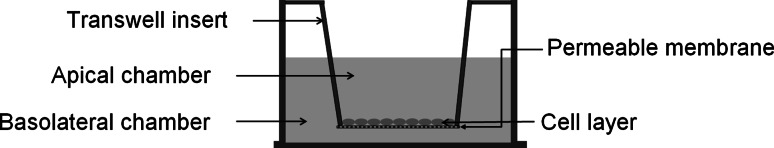
Two-compartment cell culture system contains a permeable cell culture insert, separating two compartments in a Transwell. Cells are seeded and cultured on the inserts to form a barrier between the two compartments

The skin is a barrier towards loss of water and ingress of microorganisms, UV radiation, and potentially harmful chemicals. Although the permeability of skin is an order of magnitude less than that of the intestinal epithelial cell layer, due to its large surface of almost 2 m^2^ and likelihood of dermal exposure in everyday life, the skin can pose an important absorption route for nanoparticles. The barrier function of the skin is generally attributed to its upper layer, the stratum corneum. The stratum corneum consists of stacked layers of corneocytes, enucleated flattened cells that are surrounded by impermeable cornified envelope and embedded in organized lipid bilayers. There are three potential routes by which a penetrant can diffuse across the stratum corneum: (1) across the lipid bilayers (intercellular route; Fig. 1a), (2) across the corneocytes and lipid bilayers (intracellular route; Fig. 1b), and (3) along hair follicles and sweat glands (Fig. 1c). The intracellular route is thermodynamically unfavourable due to the highly impermeable cornified envelope of the corneocytes. For most chemicals, the route across lipid bilayers represents the main diffusional pathway. The stratum corneum lipids are organized in two coexisting lamellar phases: a long periodicity phase with a repeat distance of around 13 nm and a short periodicity phase with a repeat distance of around 6 nm (Baroli 2010). The space between the tail–tail and head–head domains of the lipid bilayers (Fig. 1) restricts the size of a NP that is able to penetrate across the stratum corneum (Baroli 2010; Cevc and Vierl 2010). Another restriction factor for the penetration of nanoparticles across the intracellular route is high diffusion resistance for particles larger than 5 nm (Watkinson et al. 2013). Thus, theoretically for nanoparticles larger than approximately 5 nm the route along hair follicles might represent the predominant penetration route.

This view, however, can be challenged in the case of a compromised skin barrier, which can occur due to intrinsic and environmental factors. For instance, one of the main features of a common inflammatory skin disease, atopic dermatitis (AD), is a damaged skin barrier (Kezic et al. 2014). Furthermore, exposure to skin irritating chemicals such as detergents and organic solvents in the work place as well as in everyday life can also lead to increased skin permeability (Kezic and Nielsen 2009). Data on percutaneous penetration of nanoparticles are scarce. Recently, Labouta et al. 2011a, b showed that in contrast to intact skin barrier, the skin compromised by toluene allows for penetration of nanoparticles of 15 nm into viable skin (Labouta et al. 2011b).

## Introduction to the placental barrier

The human placenta is a unique organ, structurally complex, highly efficient, and metabolically and biosynthetically active (Aye and Keelan 2013). The placenta is responsible for the (bidirectional) transfer of substances between the maternal and foetal circulations including carbon dioxide, oxygen, water, nutrients, hormones, vitamins, and also xenobiotics including drugs and toxic compounds (Desforges and Sibley 2010). In early pregnancy, the human placenta is primarily composed of cytotrophoblasts, which continually fuse to form multinucleate syncytiotrophoblasts as pregnancy progresses. The syncytiotrophoblast consists of two polarized plasma membranes: a maternal-facing microvillous plasma membrane (MVM) and a basal plasma membrane (BM) oriented towards the foetal circulation (Kulvietis et al. 2011; Lager and Powell 2012) (Fig. 2).

The rate-limiting barrier in the human placenta for the permeation of substances between maternal blood and foetal capillaries is the syncytiotrophoblast (Young et al. 2003). Once in the cytoplasm of the syncytiotrophoblast, molecules destined for the foetus exit from the syncytiotrophoblast via the foetal facing basal plasma membrane (BM) (Desforges and Sibley 2010). At term, the placental diameter varies between 200 and 220 mm (Benirschke et al. 2006). The diffusion distance between the maternal and foetal circulations varies between 4 and 5 µm, while in the first trimester the distance varies between 50 and 100 µm (Aye and Keelan 2013; Benirschke et al. 2006). Passage across the placenta can occur via simple diffusion, pinocytosis, receptor-mediated uptake, and both active and facilitated transport (Aye and Keelan 2013). The syncytiotrophoblast plasma membranes express numerous transporters which may be regulated by foetal, maternal and placental signals (Lager and Powell 2012). The anatomy and physiology of the human placenta is different from the rodent placenta. The main difference is that in humans the syncytiotrophoblasts arise from fusion of cytotrophoblast cells and form a syncytium with no lateral cell membranes. In rodents, three trophoblast layers are present between maternal blood and foetal blood capillaries.

## Overview of currently used in vitro models to study translocation of nanoparticles

Many different in vitro models have been developed to study the translocation of nanoparticles. Most in vitro barrier models culture cells on Transwell inserts, which consist of a permeable membrane separating an apical and a basolateral compartment. Cells are seeded and cultured on the inserts to form a barrier (upon confluence of the cells) between the two compartments. Depending on the cell type selected, the Transwell model can be used to study lung, gastrointestinal, or placental transfer.

Transwells can be used to quantify both uptake of nanoparticles into the cells from the apical compartment and efflux from the cells to the basolateral compartment as a measure of translocation (Fig. 3). Fluorescent polystyrene nanoparticles are the most commonly employed because of their easy detection.

Skin in vitro models are not based on Transwell inserts, because these cell culturing models lack the principal barrier, the stratum corneum. Therefore, ex vivo skin models are mostly used for the in vitro assessment of nanoparticles translocation. Also for the other barriers, especially for the placental barrier and to a lesser extent for the lung and intestinal barrier, ex vivo models are available to test the translocation of nanoparticles.

Table 1 presents an overview of in vitro barrier models currently used to study the translocation of nanoparticles after inhalation, oral intake, dermal exposure, and across the placenta.

**Table 1:** Overview of in vitro models used to study translocation of nanoparticles

Model	Nanoparticles	Conclusion on barrier function	References
*Lung*			
A549: human type II alveolar epithelial cell line, MDM: monocyte-derived macrophages, MDDC: monocyte-derived dendritic cells	15-nm gold at the air–liquid interface	Translocation rate of 5.2 % (±4.8 %) at 4 h and 5.2 % (±5.6 %) at 24 h for 561 ng/cm^2^ and 0.5 % (±0.3 %) at 4 h and 3.95 (±3.9 %) at 24 h for 61 ng/cm^2^	Brandenberger et al. (2010)
16HBE14o-: human bronchial epithelial cell line, HUVEC: human umbilical vein endothelial cells	No nanoparticles used	16HBE14o- cells form tight junctions. The barrier function is highest in biculture compared with monocultures. Increased TEER correlated with increased occludin mRNA. Cells migrated trough inserts with 1.0-µm pores but not through inserts with 0.4-µm pores	Chowdhury et al. (2010)
A549: human type II alveolar epithelial cell line	40- and 200-nm carboxylated polystyrene nanoparticles	Nanoparticles entered cells via active energy-dependent processes. Uptake was inhibited after microtubule disruption and treatment with genistein	dos Santos et al. (2011)
RAECM: rat alveolar epithelial cell monolayers	5.3-nm quantum dots (CdSe/ZnS) with amino-conjugated, carboxylated, or non-modified surfaces	Quantum dots do not injure RAECM, and quantum dot trafficking does not appear to take place via endocytic pathways involving caveolin, clathrin, or dynamin. Translocation of quantum dots occurs both transcellularly and paracellularly	Fazlollahi et al. (2011)
NCI-H292: human bronchial epithelial cell line, Calu-3: human bronchial epithelial cell line, A549: human type II alveolar epithelial cell line	50-nm fluorescent silica nanoparticles	Calu-3 cells form tight junctions. Translocation of silica nanoparticles was 3 % in the Calu-3 cells, 9 % in the NCI-H292 cells, and 35 % in the A549 cells (cells seeded on inserts with 3.0-µm pores). The high translocation rate in A549 cells is caused by their inability to form a tight barrier	George et al. (2015)
A549: human type II alveolar epithelial cell line, Calu-3: human bronchial epithelial cell line Primary pneumocytes type II	46-nm fluorescent carboxylated and amino-conjugated polystyrene nanoparticles	Calu-3 and primary type II cells show a high TEER, whereas A549 do not. Acellular translocation of carboxylated particles was 13.5 % through inserts with 0.4-µm pores and 67.5 % through 3-µm pores. Acellular translocation of amino-conjugated particles was 4.2 % through 0.4-µm pores and 52.7 % through 3-µm pores. Calu-3 and primary type II cells showed no translocation of particles in inserts with 0.4-µm pore size. Calu-3 cells showed about 6 % translocation with 3.0-µm pore size. A549 cells were not tested because of their low TEER	Geys et al. (2006)
Primary rat alveolar epithelial cells	Quantum dots with amino-conjugated, carboxylated, or non-modified surfaces	No translocation after 24-h exposure. Disruption of the epithelial barrier causes translocation, indicative for paracellular transport	Geys et al. (2009)
A549: human type II alveolar epithelial cell line, NCI H441: human lung epithelial cell line, HPMEC: primary human pulmonary microvascular endothelial cells	No nanoparticles used. Sodium fluorescein used as permeability marker	H441 form tight junctions, A549 not. H441 showed reduced permeability compared with A549	Hermanns et al. (2004)
NCI H441: human lung epithelial cell line, A549: human type II alveolar epithelial cell line, E10: microvascular endothelial cells Culture on a chip	20-nm fluorescent polystyrene amino-conjugated nanoparticles	Physiological breathing motions increased nanoparticle transport across the lung barrier	Huh et al. (2010)
Calu-3: human bronchial epithelial cell line	Fluorescent poly(vinyl alcohol) nanoparticles	Calu-3 cells internalized up to 11 % of the applied nanoparticles. The maximum translocated fraction was 1.3 % in 14 h	Madlova et al. (2009)
A549: human type II alveolar epithelial cell line, MDM: monocyte-derived macrophages MDDC: monocyte-derived dendritic cells	15–300-nm diesel exhaust particles 20–30-nm anatase titanium dioxide nanoparticles, 20-nm single-walled carbon nanotubes	All cell types internalized the different particles	Muller et al. (2010)
A549: human type II alveolar epithelial cell line, cultured at the air–liquid interphase	9-nm cerium dioxide nanoparticles	35 % of the particles were internalized at 10 min after exposure, which increased to 60 % at 30 min and 80 % at 24 h after exposure	Raemy et al. (2011)
A549: human type II alveolar epithelial cell line, MDM: monocyte-derived macrophages, MDDC: monocyte-derived dendritic cells	20–30-nm anatase titanium dioxide nanoparticles	Titanium dioxide nanoparticles were found in all cell types as bigger membrane-bound aggregates and as single particles and smaller aggregates that were not membrane bound	Rothen-Rutishauser et al. (2008)
RAECM: rat alveolar epithelial cell monolayers	20- and 100-nm carboxylated, sulphated, or aldehyde-sulphated and amino-conjugated polystyrene nanoparticles	Translocation was 20–40 times faster for positively charged particles compared with negatively charged particles of the same size. Translocation decreased with increasing particle size: 20-nm particles were transported about 3 times faster compared with 100-nm particles	Yacobi et al. (2008)
3D human airway model, cultured at air–liquid interface	13.8-nm cerium dioxide; inverse agglomeration size in 3D medium with dose	Droplet exposure: translocation showed inverse dose–response when expressed as percentage of dose applied	Frieke Kuper et al. (2015)
*Gut*			
Caco-2 and MTX-E12 monolayers	Hydrophobic polystyrene, bioadhesive chitosan, and stealth PLA-PEG nanoparticles	Mucus is presenting a major barrier for hydrophobic polystyrene NP and chitosan. Chitosan NP seemed to be taken up by adsorptive transcystosis polystyrene NP via non-adsorptive transcystosis	Behrens et al. (2002)
Caco-2 +M cells	Ag nanoparticles 20, 30, 60, 110 nm	Study focussed on gene expression. No Ag NP-specific differential gene expression was noted. Very limited (1 %) translocation	Bouwmeester et al. (2011)
Caco-2	Latex 2 µm particles	In vivo uptake after early time points after single dose at villous and not Peyer’s patch. While M cell model has looser tight junctions than Caco-2 cells, uptake level of particles is comparable	Carr et al. (2012)
Caco-2 + M cells (Raji)	Carboxylated latex nanoparticles 200 nm	50-fold higher translocation in co-cultures (8.0e−4 % transported) compared with monocultures	des Rieux et al. (2007)
Caco-2 + M cells	200-nm and 500-nm polystyrene nanoparticles carboxylated or amino-conjugated	Amino-conjugated 200-nm nanoparticles were translocated more (0.002 %) than carboxylated nanoparticles. Absence of serum resulted in increased translocation. 500-nm nanoparticles were not translocated	(des Rieux et al. 2005)
Caco-2 cell rat ileum (ex vivo), and systemic biodistribution after oral gavage	Rhodamine-B-labelled carboxylated chitosan grafted nanoparticles (RhB-CCNP) (300, 600, and 1000 nm) and similar Zeta potentials (−35 mV)	RhB-CCNP-BSA with smaller sizes (300 nm) demonstrated elevated intestinal absorption, compared with the others	He et al. (2012)
Caco-2 + HT29-MTX	Insulin-loaded chitosan nanoparticles	CSK peptide modification showed enhanced transport. The presence of mucus increased the translocation of both modified and unmodified nanoparticles	Jin et al. (2012)
Caco-2 + M cells	Chitosan-DNA nanoparticles	Ligand decoration has the most dramatic effect on the transcytosis rate: transferrin modification enhances transport through both models by three- to fivefold. Transport through the M cell co-culture model is fivefold that of the intestinal epithelial monolayer, with at least 80 % of the chitosan-DNA nanoparticles taken up in the first 30 min	Kadiyala et al. (2010)
Caco-2 + HT29-MTX + M cells	50- and 200-nm polystyrene nanoparticles, carboxylated, amino-conjugated, or plain	Co-exposure of monolayers to either 50 or 200-nm nanoparticles and iron-ascorbate solution resulted in increased translocation of iron 50-nm NPS translocated equal in both co- (Caco and HT29) and triculture models. 200-nm nanoparticles translocated significantly more in tri cultures, only at 37 °C at 37 and 4 °C	Mahler et al. (2012)
Caco-2	Polyelectrolyte complex nanoparticles of spermine (SPM) with polyacrylic acid (PAA) polymer	Permeation enhancing effects following exposure to nanoparticles were associated with a reversible decrease in TEER values, suggesting a paracellular permeation pathway by reversible opening of the tight junctions Higher permeation enhancing profiles of SPM-PAA nanoparticles, as compared with SPM solution or PAA nanoparticles prepared by ionic gelation with MgCl_2_ (Mg-PAA nanoparticles)	Makhlof et al. (2011)
Caco-2 with and without HT29-MTX with and without M cells	50- and 100-nm polystyrene nanoparticles, carboxylated, amino-conjugated, or plain	The incorporation of mucus in the model reduced the translocation of 50-nm nanoparticles compared with Caco-2 only. The incorporation of M cells resulted in a translocation levels close to those as observed for the monoculture. The surface charge of the NP was very important for the (changes) in translocation in the different models. 100-nm nanoparticles were translocated at a very low level	Walczak et al. (2014)
Caco-2 with HT29-MTX coupled to in vitro digestion	50 polystyrene nanoparticles, carboxylated, amino-conjugated, or plain	Incubation of the nanoparticles in the in vitro digestion model resulted in increased translocation levels compared with ‘non-digested’ nanoparticles	Walczak et al. (2015)
*Ex vivo* mice intestinal tissue in Ussing chambers	Hydrodynamic size of 130-nm slices exposed for 2 h to 50 μg/ml	Local Ti spots in tissues were observed, increased permeability of 4 kDa dextran after Ti NP exposure. No Ti translocation could be detected	Brun et al. (2014)
*Ex vivo* porcine intestinal tissues in Ussing chambers	50- and 200-nm polystyrene nanoparticles exposed for 4 h to 100 μg/ml	Entrapment of nanoparticles in superficial part of the tissue was observed. Translocation not determined	Schimpel et al. (2014)
*Skin*			
Full thickness human skin, handmade vertical diffusion cell	Fe oxide, <10 nm	Nanoparticles were able to penetrate into viable skin layers	Baroli (2010)
Human, previously frozen and viable skin, Franz diffusion cell	Au 6 and 15 nm	6-nm gold nanoparticles in toluene penetrated into epidermal layers of human skin	Labouta et al. (2011b)
Porcine skin, home-made static diffusion cell	TiO_2_, >50–100 nm	TiO_2_ was not able to penetrate into viable skin layers even when the skin was previously compromised by physical or mechanical constrains and/or solar radiation	Miquel-Jeanjean et al. (2012)
Porcine skin, flow-through diffusion cell	TiO_2_ and ZnO 60–200 nm present in the sunscreen formulation	UVB-sunburned skin enhanced penetration of the TiO_2_ or ZnO NP. TiO_2_ and ZnO NP penetrated into the viable skin layers but not in receptor fluid	Monteiro-Riviere et al. (2011)
Full thickness human skin, Franz and flow-through diffusion cell	Quantum dots 13–29 nm with different surface modifications	Differences in penetration depended on the surface coatings of the NP. No penetration at the physiological pH, but at slightly basic pH, nanoparticles did penetrate into viable skin layers	Prow et al. (2012)
*Placenta*			
BeWo b30 Transwell model	Dexamethasone-loaded poly(d,l-lactide-coglycolide) (PLGA) nanoparticles (146 and 232 nm)	Transport across the placental barrier of dexamethasone-loaded PLGA nanoparticles was influenced by size 2 h Papp values: 6.0 × 10^−5^ ± 1.6 × 10^−5^ cm/s (146 nm); 4.8 × 10^−5^ ± 1.6 × 10^−5^ cm/s (232 nm)	Ali et al. (2013)
BeWo b30 Transwell model	Polystyrene nanoparticles 50 and 100 nm (Fluoresbrite; Polysciences)	3.5 % (50 nm) and 0.6 % (100 nm) of the initial amount added to the apical chamber was found in the basal chamber after 24 h 24 h Papp values: 3.9 ± 0.5 × 10^−5^ cm/s (50 nm); 9.7 ± 9.4 × 10^−6^ cm/s (100 nm)	Cartwright et al. (2012)
BeWo b30 Transwell model	Iron oxide and silica nanoparticles (23–38 nm)	Nanomegnetite Na-oleate-coated (OC-Fe3O4) and Fluorescent rhodamine-labelled silica (Fl-SiO2) nanoparticles are able to cross the BeWo b30 barrier. Fl-SiO2 transport (24-29%) after 6 h was unaffected by size 6 h Papp values: 0.017 ± 0.002 cm/s (OC-Fe3O4); 0.018 ± 0.007 cm/s (Fl-25 SiO2); 0.017 ± 0.009 cm/s (Fl-50 SiO2)	Correia Carreira et al. (2013)
BeWo b30 Transwell model	Polystyrene nanoparticles of 50 nm	Limited transport (3-15%) of 50 nm polystyrene nanoparticles with positive and negative charge after 24 h. 24 h Papp value: 0.3 × 10^−6^ cm/s (amino-conjugated polystyrene-NP); 13 × 10^−6^ cm/s (carboxylated polystyrene-NP)	Kloet et al. (personal communication)
BeWo (ATCC clone) Transwell model	PEGylated gold nanoparticles of 10–30 nm	Internalisation of PEGylated gold nanoparticles of 10 nm in BeWo cells up to 48 h after exposure as analysed by TEM	Myllynen et al. (2008)
BeWo b30 Transwell model	Rhodamine labeled silica nanoparticles of 25 nm	Limited transport of 25 nm silica nanoparticles 24 h Papp value: 1.5 × 10^−6^ ± 1.56 × 10^−6^ cm/s	Sonnegaard Poulsen et al. (2013)
Ex vivo human placenta perfusion model	PEGylated gold nanoparticles of 10–30 nm	No detection of PEGylated gold nanoparticles of 10–15 nm on fetal side	Myllynen et al. (2008)
Ex vivo human placenta perfusion model	Polystyrene (polystyrene) 50, 240 nm (Kisker GbR, Steinfurt, Germany). 80, 500 nm (Polyscience Europe GmbH, Eppelheim, Germany)	polystyrene beads up to a diameter of 240 nm were taken up by the placenta and able to cross the placental barrier Perfusion with 25 μg/mL 50-nm polystyrene beads in the mater¬nal circuit. The level of particles measured after 180 min was 8.90 ± 1.80 μg/mL in the fetal perfusion medium and 18.1 ± 5.64 μg/mL in the maternal perfusion medium	Wick et al. (2010)
Ex vivo human placenta	PAMAM dendrimers of 5–6 nm	PAMAM dendrimers of 5-6 nm crossed the perfused human placenta in relatively small amounts within 6 h	Menjoge et al. (2011)
Ex vivo human placenta perfusion model	Fluorescently labelled polystyrene particles with sizes of 80 and 500 nm	The 80 nm particles were able to cross the placental barrier and provide a perfect example for a substance which is transferred across the placenta to the foetus while the 500 nm particles were retained in the placental tissue or maternal circuit	Grafmuller et al. (2013)
Ex vivo human placenta perfusion model	Ex vivo human placenta perfusion model	4.2 and 4.6 % for 25 and 50 nm silica nanoparticles reached the fetal perfusate after 6 h	Sonnegaard Poulsen et al. (2013)

## Lung in vitro models

The most frequently used lung epithelial cells lines are A549, Calu-3, H441, and 16HBE14o-. Of these cell lines, Calu-3, H441, and 16HBE14o- form tight junctions, but A549 do not (Lehmann et al. 2011; Chowdhury et al. 2010, Geys et al. 2006, Hermanns et al. 2004, George et al. 2015). In in vitro translocation studies, primary rat alveolar cells have also been frequently used (Fazlollahi et al. 2011; Geys et al. 2009; Yacobi et al. 2008).

Besides differences in cell types, in vitro lung barrier models differ in whether they are submerged or cultured at the air–liquid interface. Submerged models have the advantage of being technically simple. However, the culture medium can alter the properties of the nanoparticles, and subsequently their uptake and effects. Air–liquid models mimic more realistically the inhalation exposure; therefore, many air–liquid models have been developed recently (Blank et al. 2006; Brandenberger et al. 2010; Frohlich et al. 2013; Herzog et al. 2013; Holder and Marr 2013; Lenz et al. 2009, 2013; Raemy et al. 2011; Rothen-Rutishauser et al. 2009; Savi et al. 2008; Xie et al. 2012). Disadvantages are the complexity of the system to maintain constant temperature and humidification, and the high costs in comparison with submerged models.

In recent years, co-culture models containing more than one cell type are used to mimic the lung barrier more closely compared with monocultures (Klein et al. 2011). Most models use lung epithelial cells as a basis, those being either primary cells or immortalized cell lines. To obtain co-culture models, different cell types are added to the basic model. The first type of co-culture models includes, in addition to epithelial cells, endothelial cells to mimic the alveolar-capillary barrier (Bermudez et al. 2002; Chowdhury et al. 2010; Hermanns et al. 2010; Hermanns et al. 2004; Papritz et al. 2010). This type of model can be extended by the addition of alveolar macrophages, mast cells, and/or type II alveolar cells. The second type of models does not include endothelial cells, but adds dendritic cells and macrophages to the epithelial cell layer (Brandenberger et al. 2010; Muller et al. 2010; Rothen-Rutishauser et al. 2008; Rothen-Rutishauser et al. 2005). This type of model can also be completed with type II alveolar cells. The third type of models includes, in addition to the epithelial cells, fibroblasts instead of endothelial cells, which can be extended by adding dendritic cells.

A limited number of nanoparticles have been studied to test translocation across the in vitro lung barrier. These include polystyrene nanoparticles, titanium dioxide nanoparticles, quantum dots, cerium dioxide nanoparticles, gold nanoparticles, silica nanoparticles, diesel particles, and single-walled carbon nanotubes (Table 1). The translocation rate of nanoparticles is higher in models with cells that do not form tight junctions compared with cells that do (Geys et al. 2006; Hermanns et al. 2004; George et al. 2015). Probably, the higher translocation rate is a consequence of paracellular transport, which will not occur in a healthy lung but might occur in a damaged or inflamed lung. The pore size of the permeable membrane of the inserts also influences the translocation rate of nanoparticles: the larger the pore size, the higher the translocation rate (Geys et al. 2006). Therefore, for each single type of nanoparticles, the translocation across the different types of inserts should be tested without cells to assess whether the nanoparticles are not withheld by the insert itself.

Ex vivo tissues as model for the lung are not addressed here. The precision-cut lung slices (PCLS) taken from human and rodent lungs have been used to study translocation and toxicity of nanomaterials. The use of PCLS for translocation of nanoparticle-mediated drug delivery has recently been reviewed by Paranjpe and Muller-Goymann (Paranjpe and Muller-Goymann 2014).

## Gut in vitro models

Orally ingested nanoparticles are exposed to continuously changing conditions while transiting through the gastrointestinal tract, which influences their nature and characteristics (Bellmann et al. 2015). In vitro GI models aim to mimic the gastrointestinal environment as closely as possible, to generate physiologically relevant results. These models focus either on the aspects of dynamically changing GI conditions during digestion by simulation of the transit of nanoparticles along the GI tract from the mouth towards the large intestine (digestion models), or on mimicking translocation and uptake behaviour (in vitro human intestinal epithelium models) (Lefebvre et al. 2014).

Human digestion models can first be simple and static, and mimic only gastric or small intestinal conditions, in which materials are incubated with simulated gastric fluids, simulated small intestinal fluids or buffers at static pH values (Mwilu et al. 2013; Minekus et al. 2014). More complex static models often include most of the relevant GI conditions, i.e. the oral, gastric, small intestinal (and large intestinal conditions) (Oomen et al. 2003; Van de Wiele et al. 2007; Versantvoort et al. 2005). Recently, these models have been used to assess the fate of 60-nm silver nanoparticles, and nanometre-sized silica (synthetic amorphous silica) nanoparticles during digestion (Peters et al. 2012; Walczak et al. 2013). In contrast to static models, more complex dynamic models simulate successive changes in conditions (i.e. pH, secretion of digestive fluids) and transit times (Helbig et al. 2013; Kong and Singh 2010; Minekus et al. 1995; Wickham et al. 2009; Zangenberg et al. 2001). Such a dynamic computer controlled model was used to study the behaviour of engineered nanoclay materials (Newsome 2014).

Translocation and uptake of nanoparticles can be addressed by cellular models that can also be divided in relatively simple models or more complex ones (Lefebvre et al. 2014). Amongst the variety of cell models that are available in vitro, intestinal Caco-2 cells (human epithelial colorectal adenocarcinoma cells) are the most commonly used cell type (Miret et al. 2004) in nanoparticle translocation studies. Caco-2 cells are regarded as model cells for enterocytes, the most abundant epithelial cell type in the intestine. Several examples can be found in the literature where monocultures of Caco-2 cells have been used to study the in vitro translocation of nanoparticles, (mainly polystyrene, but also silicon, silver, and organic nanoparticles) (Bhattacharjee et al. 2013; des Rieux et al. 2007; des Rieux et al. 2005; Mahler et al. 2009; Natoli et al. 2012; Nkabinde et al. 2012; Walczak et al. 2014).

A potential drawback of Caco-2 monolayers is the lack of a mucus layer (at least in conventional models), which can, however, be introduced by co-culturing Caco-2 cells with HT29-MTX cells (human colon adenocarcinoma mucus-secreting cells) (Behrens et al. 2002; Mahler et al. 2009; Scaldaferri et al. 2012; Walczak et al. 2014). The mucus layer consists of mucin glycoproteins that form viscoelastic gels, and it is thinner in the small intestine compared with the large intestine. Though this makes the small intestine a prominent place for nanoparticle uptake, the mucus layer also represents a hindrance allowing selective passage of materials. The mucus can entrap nanoparticles (and thus reduce their translocation) because it poses a physical barrier due to its thickness, density, negative charge, and constant renewal (Cone 2009; Crater and Carrier 2010; Szentkuti and Lorenz 1995). In addition, mucus has a protective function for bio-relevant fluids present in the lumen of the gut, and direct exposure of Caco-2 cells (without a mucus layer) to lumen content stimulants reduces the barrier function of Caco-2 cells models (Ingels et al. 2002). This is probably due to the lack of mucus layer making the Caco-2 cells much more sensitive to direct exposure to the low pH and high osmolality of the buffers (Westerhout et al. 2014).

To further increase the complex anatomy of the human gut epithelium, human intestine microfold (M) cells are introduced in epithelial monolayers. While M cells in total compose less than 1 per cent of the small intestine epithelial cell layer, they are responsible for the uptake and translocation of relatively larger particles (Antunes et al. 2013; Bouwmeester et al. 2011; des Rieux et al. 2007; des Rieux et al. 2005; Kerneis et al. 1997; Martinez-Argudo et al. 2007; Walczak et al. 2014). Recently, in vitro digestion models have been linked to in vitro gut epithelial models to study first the digestion of nanoparticles and next the bioavailability of the digested particles in the intestines (Walczak et al. 2015) (see Table 1).

Several ex vivo gut models have been developed to study the translocation of chemicals. Different approaches are used ranging from in situ perfusion (intestinal loop) models to models in which part of the gut epithelium is excised from animals and maintained in, for example, Ussing chambers for a limited period of time. These models have recently been reviewed by Lefebvre et al. (2014). These authors summarize studies in which the translocation of mainly organic nanoparticles have been evaluated, only a limited number of studies used polystyrene or titanium nanoparticles that have also been used in vitro (see Table 1).

## Skin in vitro models

For the determination of dermal absorption of chemicals, several guidelines have been established by prescribing the type of skin membrane, species, and experimental protocol (OECD 2004). However, specifically for nanoparticles, there are no such guidelines and critical evaluation of the current models is missing. Skin in vitro models differ in the type of the skin membrane (full thickness skin vs. dermatomed skin), species (human vs. animal skin), vehicle and type of the diffusion cell, which hampers comparison and interpretation of the results.

**Fig. 4:**
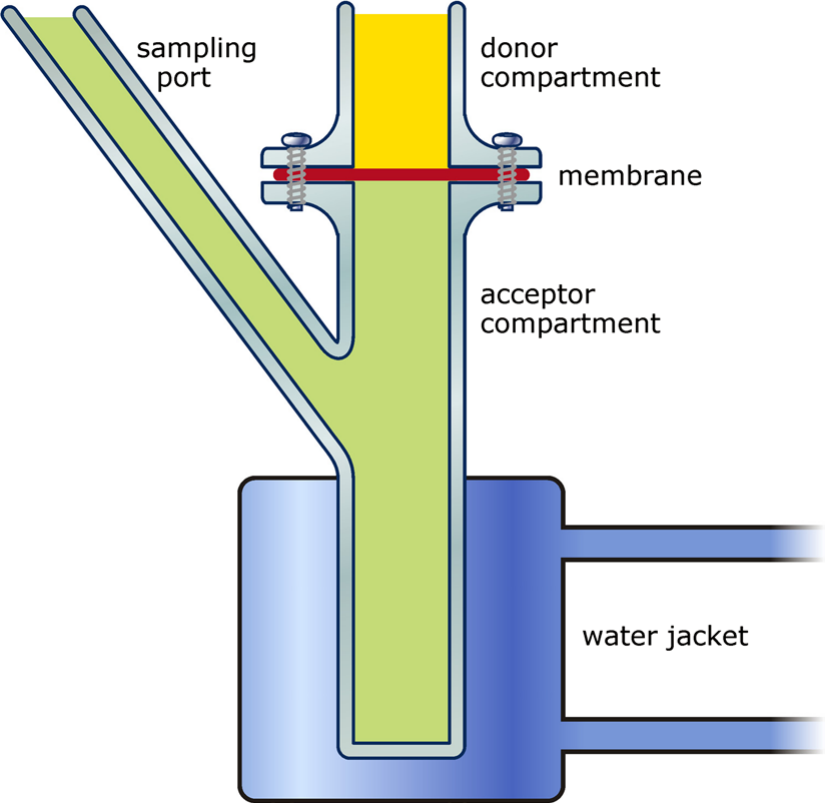
In vitro diffusion chamber to test bioavailability of nanoparticles across the skin barrier

In contrast to the lung, intestinal and placental barrier, no Transwells are used for percutaneous penetration studies. The reported in vitro studies on percutaneous penetration of nanoparticles have been performed by using either a Franz static cell or a flow-through diffusion cells. In both systems, the skin membrane is clamped between two chambers, one of which contains a vehicle supplemented with the investigated chemical (donor chamber) and the other one a receptor fluid from which the penetrated chemical will be sampled (Fig. 4) (Jakasa and Kezic 2008). In several studies, human reconstructed skin models have been applied although there were concerns regarding a less well-developed barrier in these models and the absence of the follicular penetration route that might play an important role for translocation of nanoparticles (Labouta et al. 2011a, b). In in vitro assays, usually a cryopreserved skin is used, which might, however, lead to changes of the skin barrier and shrinkage of the hair follicles (Labouta et al. 2011a).

Although human skin is regarded as a gold standard, a large number of studies on percutaneous penetration of nanoparticles use different animal models including mice, rat, and pig skin (Labouta and Schneider 2013). However, structural and morphological differences between human and animal skin, especially concerning the density of the hair follicles, thickness of the skin layers, skin lipid composition, and structure, could certainly affect the NP percutaneous penetration (Labouta and Schneider 2013). Another problem encountered by using hairy animals is damage of the skin barrier due to hair clipping.

## Placenta in vitro models

Models for studying transplacental transport have long been based on the perfused isolated human placenta studied in ex vivo study designs. The perfused isolated human placenta ex vivo model presents a directly relevant alternative that maintains the complexity of the intact placenta (Saunders 2009). Transport studies in the ex vivo intact placenta are technically challenging and require large quantities of substances to be tested. Therefore, models using representative placental cell lines in Transwell study designs are being developed as in vitro alternatives. Most commonly used human placental cell lines are the BeWo, Jar, and JEG-3 cell lines, which have been applied to study transplacental transfer of a variety of drugs and compounds. The most popular cellular model consists of the BeWo cell line, representing a choriocarcinoma-derived placental cell line that strongly resembles cytotrophoblastic cells. The BeWo b30 subclone can be grown on Transwell inserts to form confluent cell layers, enabling the quantification of both uptake into the cells from the apical compartment (maternal side) and efflux from the cells to the basolateral compartment (foetal side) (Buerki-Thurnherr et al. 2012). In some studies these models have been validated by comparison of the transport rate across the Transwell cellular BeWo b30 layer and the transport rates detected in ex vivo placental models for the same compounds (Li et al. 2013; Poulsen et al. 2009).

Although the type and nature of the nanoparticles studied in in vitro placental models are different and the number of studies is limited, some general observations can be made. Nanoparticles can be transported across the placental barrier, where their size and type of nanoparticles influence the efficiency of the transport. Furthermore, in contrast to drugs and other xenobiotic bulk chemicals, transport of several nanoparticles across the placental barrier appears to be highly variable. The latter conclusion can be derived from the observation that for the nanoparticles studied so far the amount (%) of the nanoparticles transported across the BeWo b30 cell layer varies from 0.6 to 29 % in 6 h (Table 1). Comparison of this transport rate to that reported in the BeWo model for several chemicals mounting up to 30 % in 2 h reveals that the transport of nanoparticles across the BeWo cell layer, just as that of chemicals, may be limited or significantly depending on the type of nanoparticles. This implies that a validated in vitro model to quantify the potential transport of nanoparticles across the placental barrier would be of high value to set priorities for further in vivo testing, thereby avoiding in vivo testing of all newly developed nanoparticles.

## General remarks on currently used in vitro models to study translocation of nanoparticles

Currently used in vitro models to study translocation of nanoparticles are mostly based on cell culturing on Transwell inserts or on the use of ex vivo tissues. Transwell inserts can differ in the type of plastic they are made of and in the pore size of the permeable membrane. These differences between the Transwell inserts influence the translocation rate (Cartwright et al. 2012; Geys et al. 2006). Transwell inserts with a larger pore size give increased translocation rates compared with inserts with smaller pore sizes. In addition, some types of plastic of the Transwell inserts yield decreased translocation due to nanoparticle adsorption on the material surface. Therefore, for each single type of nanoparticles, the translocation across the different types of inserts should be tested without cells to assess whether the nanoparticles are not withheld by the insert itself.

Next to this, different cell types are used, which clearly influences the translocation and the mechanism of translocation. When cell types lack the formation of tight junctions, nanoparticles can easily pass between the cells resulting in a higher translocation rate compared with cell types that do form tight junctions. To prevent paracellular transport, epithelial cells forming tight junctions are preferred. As already discussed, the incorporation of a mucus layer in gut epithelial models decreases the translocation (Walczak et al. 2014). This mucus layer might be a very important barrier for nanoparticles in vivo because of electrostatic repulsion (for negatively charged nanoparticles) and mucus entrapment (for positively charged nanoparticles) (Husain et al. 2001; Lai et al. 2007; Norris et al. 1998; Szentkuti and Lorenz 1995). Similarly, such a mucus layer might also be essential for in vitro lung barrier models, since nanoparticles first come in contact with the pulmonary surfactant if they are deposited in the lungs. Lastly, the use of specific cells types in co-culture on top of an epithelial layer can alter the translocation rate of the nanoparticles. For instance, alveolar macrophages in lung models can decrease the translocation rate by uptake of nanoparticles, while M cells in intestinal models can enhance translocation. The addition of these specific cell types can improve the in vitro models by more closely mimicking the in vivo situation in which, for example, the lungs are protected from particles via phagocytosis by alveolar macrophages.

Some reported in vitro models use primary cells, while others use immortalized cell lines. On one hand, primary cells have a more differentiated phenotype compared with cell lines. On the other hand, the isolation of primary cells is often experimentally challenging: the cells dedifferentiate after isolation, differ from batch to batch, and proliferate to a limited extent. Cell lines are easy to work with, well characterized and more homogenous, but they show only few characteristics of differentiated cells. Overall they only poorly represent the in vivo situation (Hartung et al. 2002; Klein et al. 2011). In in vitro lung translocation studies, primary rat alveolar cells have been frequently used (Fazlollahi et al. 2011; Geys et al. 2009; Yacobi et al. 2008). Recent, biologically complex human intestinal tissues have been cultured using human (induced) pluripotent stem cells, resulting in human intestinal organoids that have secretory and absorptive functions (Brugmann and Wells 2013). Comparable systems have now been modified into mature epithelial, functional and polarized monolayers grown on Transwell membranes, which are suitable for translocation studies (VanDussen et al. 2014).

*Ex vivo* models maintain the complexity of the physiological barriers (Saunders 2009). However, transport studies in ex vivo models are technically challenging, time consuming, and can require large amounts of the test substance.

Advances in the field of miniaturized and microfluidic devices have recently led to the concept of organ-on-a-chip models (Bhatia and Ingber 2014; Huh et al. 2012; Moraes et al. 2012; van der Meer and van den Berg 2012), which can be seen as hybrid devices combining cells and microfabricated structures aiming to recapitulate the dynamic physical, cellular, and functional features of human tissues (Ardavin et al. 2001; Huh et al. 2010. 2013; Schimek et al. 2013; Wagner et al. 2013). These devices first provide a high control on the cell microenvironment (e.g. physical and chemical parameters) together with dynamic culture conditions, since they are embedded in a microfluidic format (Whitesides 2006). Furthermore, these devices can include active elements, which allow exposing cells to mechanical stimuli and surface strains, by stretching the substrate on which they are grown (Sinha et al. 2015). These approaches mostly rely on 3D cell culture conditions, which are acknowledged to better mimic the in vivo conditions compared with conventional monolayer models (Harink et al. 2013).

Over the years, examples of organ-on-a-chip models in the literature have diversified and, for instance, include gut (Kim and Ingber 2013), lung (Huh et al. 2007, 2010; Nalayanda et al. 2010), or even blood–brain barriers (Griep et al. 2013; Huh et al. 2013; Wolff et al. 2015). These models can be of particular interest to the field of nanotoxicology. Huh et al., for instance, have developed a complex lung-on-a-chip model, which recapitulates movements associated with breathing that is accompanied by cyclic stretching of cells (Huh et al. 2010). This device has been applied for NP testing, showing that breathing motion is essential for such nanotoxicological assays. The same group, using the same principle, later proposed a gut-on-a-chip model integrating peristaltic motion associated with digestion. Interestingly, exposed to a combination of dynamic culture and mechanical strain, Caco-2 cells were producing mucus and microvilli features were formed (Kim and Ingber 2013). Finally, organ-on-a-chip devices are amenable to parallelization and automation; they are easily coupled to virtually any detection technique, and different organ models can even be combined on one device (Wagner et al. 2013) to eventually mimic the whole body/organism. The availability of such complex and in vivo-like models is expected to facilitate the implementation of the 3R’s legislation regarding animal experimentation (Marx et al. 2012; van de Stolpe and den Toonder 2013).

## Accuracy of current in vitro translocation models compared to in vivo data

In vitro translocation studies show mostly limited transport of nanoparticles across the lungs, gut, skin, and placenta. In the sections below, we discuss the comparison between in vitro data and available in vivo data. We included in vivo studies that measured the bioavailability of similar nanoparticles compared to their in vitro counterparts. However, most in vivo data come from separate studies in which not exactly the same type of nanoparticle is used: for instance, the nanoparticles differ in size. Another marked difference is that the in vitro models are composed of cells (often cell lines) with a human origin, while the in vivo models mostly are rodents. Therefore, we first compared data from studies using nanoparticles of the same chemical composition although they sometimes differed for other characteristics. In Table 2, we included studies that used exactly the same nanoparticles in both an in vivo and an in vitro design.

**Table 2:** Comparison in vitro and in vivo translocation of nanoparticles

Nanoparticle	In vivo study design	In vivo translocation	In vitro study design	In vitro translocation	Comparison	References
*Intestine*						
Polystyrene	Single oral administration of 50-nm nanoparticles with different surface modifications	Oral bioavailability was estimated to range between 1.7 and 0.2 % of administered dose. Not all tissues were harvested	Monolayers of co-cultures of Caco-2 and or HT29-MTX were exposed to nanoparticles for 24 h	Translocation was determined to the basolateral compartment. Translocation ranged between 12.3 and 1.6 %	The same nanoparticles were used in these studies. Duration of exposure was different. The in vitro models overestimated the estimated in vivo bioavailability	Walczak et al. (2014)
TiO_2_	Hydrodynamic size of 130-nm single oral gavage to 12.5 mg/kg of TiO_2_ nanoparticles dispersed in 150 μl of water	After 6 h tissues were harvested, Ti was detected in gut tissues but very low and could not be quantified due to the limit of detection of the used methods (1-2 ppm in μXRF)	Monolayers of Caco-2, and or HT29-MTX and or M cells were exposed to 50 μg/mL TiO_2_ nanoparticles for 48 h	TiO_2_ could not pass the polyester membranes No TiO_2_ below the basolateral membrane of Caco-2 or Caco-2/H29-MTX co-cultures was observed. Incorporation of M cells resulted in basolateral transport of TiO_2_	Both in vivo and in vitro experiments indicated translocation of TiO_2_. Due to low sensitivity of the detection techniques this could not be quantified	Brun et al. (2014)
TiO_2_	Single oral administration of 18-nm TiO_2_ at a dose of 100 mg/kg	After 24 h tissues (including small intestine and caecum), but no increased in Ti levels were observed	Monolayers of Caco-2 cells were exposed to 100 μg/ml TiO_2_ NPs for 48 h	Translocation at the limit of detection was observed (i.e. 0.1 ppm, equivalent to 0.4 % translocation)	*In vitro* very low translocation was shown, in vivo this was not detected after a single oral administration	Janer et al. (2014)
TiO_2_	Rats were exposed to a single dose of 5 mg/kg different types of TiO_2_ (mean particle size 40 nm, 40-50 nm, 120 nm, and up to 5 um)	Up to 96 h post-administration no translocation of Ti was detected to blood, several organs, and urine	Monolayers of Caco-2 and M cells were exposed for 24 h to 250 μg/ml TiO_2_ nanoparticles	No translocation could be detected	No translocation could be detected	MacNicoll et al. (2015)
*Lung*						
Silver	Intratracheal instillation in ICR mice of AgNO_3_ or silver nanoparticles of 20 nm	After 4 h, 7 % of the initial dose of Ag was recovered in the liver in AgNO_3_-treated mice, and only a trace amount of Ag was detected in the liver in Ag nanoparticles-treated mice. The concentration of Ag in the lung tissue was significantly higher in the Ag nanoparticles-treated mice compared with the AgNO_3_-treated mice	J774.1 murine macrophage cells exposed to AgNO_3_ or silver nanoparticles of 20 nm, 60 nm, and 100 nm for 24 h	Both AgNO_3_ and Ag nanoparticles were taken up by J774.1 cells. Ag in the AgNO_3_-treated cells was bound to metallothioneins, whereas Ag in the Ag nanoparticles-treated cells was not. The Ag nanoparticles were agglomerated and accumulated in the lysosomes. Uptake was not quantified	In vivo, Ag ions appeared to translocate from the lungs to other tissues rapidly. *In vitro*, Ag from AgNO_3_ binds to metallothioneins, whereas Ag nanoparticles accumulate in lysosomes	Arai et al. (2014)
Multi-walled carbon nanotubes	Oropharyncheal aspiration in C57BL6 mice of uncoated and aluminium-coated MWCNT of 0.5–40 µm length	MWCNT-containing macrophages were found in the alveolar region and around the airways of the lower lung. Tissue distribution was not measured	THP-1 human macrophages and primary human monocytes were exposed to uncoated and aluminium oxide-coated MWCNT for 24 h	Both coated and uncoated MWCNT were taken up by THP-1 cells. Uptake was not quantified and no translocation measured	Both in vivo and in vitro, MWCNTs are taken up by alveolar macrophages	Taylor et al. (2014)
*Skin*						
Titanium dioxide	Pig skin exposed for 48 h to coated TiO_2_ particles 14–16 nm	Most of Ti found in the upper layers of the stratum corneum, however, slight Ti above background has been found in the epidermis and superficial papillary dermis. The levels of TiO_2_ were higher in UV-treated skin	Porcine skin, flow-through diffusion cell, exposure duration 24 h	TiO_2_ remains mainly in the stratum corneum. Although TiO_2_ nanoparticles have been detected in the epidermis and dermis, there was no penetration into receptor fluid	Both in vitro and in vivo show that the penetrated TiO_2_ resided primarily in the upper layers of the stratum corneum, although slight penetration into viable layers occurred	Monteiro-Riviere et al. (2011)
*Placenta*						
Gold	Ex vivo human placental model exposed to PEGylated gold nanoparticles for 1 or 6 h	PEGylated gold nanoparticles of 10, 15, and 30 nm were not observed in the foetal part of the placenta	BeWo cells exposed to PEGylated gold nanoparticles	Internalization of PEGylated gold nanoparticles of 10 nm in BeWo cells up to 48 h after exposure as analysed by TEM	In vitro, there was internalization of gold nanoparticles in cells of the placenta, while ex vivo no gold nanoparticles were observed in the foetal part of the placenta	Myllynen et al. (2008)

The table shows studies in which similar nanoparticles are tested both in vivo and in vitro

## Lung barrier

The translocation rate between in vitro lung barrier models and in vivo inhalation studies differs depending on the tested nanoparticles (Table 2). In addition, some studies determined the cellular uptake and not the translocation rate of nanoparticles. The information on their localization can also be used in the comparison between in vitro and in vivo data.

For the most frequently used polystyrene nanoparticles, the translocation rate, both in vitro and in vivo, is very low 1 day after exposure. Depending on the pore size of the inserts, the translocation amounts to 0–6 % in vitro against 0.05–2 % in vivo. However, one in vivo study shows accumulation over time of polystyrene nanoparticles in the thymus (Sarlo et al. 2009), which cannot be demonstrated in an in vitro lung model. One in vitro study reported a size-dependent translocation rate (Yacobi et al. 2008), which is in line with the size-dependent distribution shown after oropharyngeal aspiration (Sarlo et al. 2009). Also for gold nanoparticles, the in vitro translocation rate ranges from 0.5 to 5.2 % (Brandenberger et al. 2010). For comparison this can be compared to the in vivo translocation to the liver of 0.7 to 2.8 %; clearly, this is an underrepresentation of the complete systemic availability (Sadauskas et al. 2009; Sung et al. 2011; Takenaka et al. 2006; Yu et al. 2007). For cerium dioxide, quantum dots, silica, and titanium dioxide nanoparticles, different translocation rates are found in vitro and in vivo. For cerium dioxide, the uptake rate in vivo is very low, and only about 10 % of the inhaled cerium dioxide was detected in the lungs (Geraets et al. 2012), while 80 % of the particles was internalized in cells in vitro (Raemy et al. 2011). After inhalation exposure, quantum dots could be detected in the liver and kidney at relatively high amounts of 15 and 5 %, respectively (Ma-Hock et al. 2012). However, in vitro, no translocation of quantum dots across primary rat alveolar epithelial cells was observed (Fazlollahi et al. 2011; Geys et al. 2009). Silica nanoparticles were able to translocate across monolayers of Calu-3, NCI-H292, and A549 cells in vitro (George et al. 2015), but were not detected in tracheobronchial lymph nodes after inhalation exposure (Arts et al. 2007). However, the silica content was not measured in any other tissue or organ besides the lung and lymph nodes, so there might have been some in vivo translocation to the blood, liver, and other organs. Finally, titanium dioxide nanoparticles were internalized by A549 epithelial cells, monocyte-derived macrophages, and monocyte-derived dendritic cells in vitro (Muller et al. 2010; Rothen-Rutishauser et al. 2008), while the in vivo studies showed that most nanoparticles retained in the alveolar space in the lung-lining fluid directly after exposure, and were found later mostly inside alveolar macrophages (Creutzenberg et al. 2012; Geiser et al. 2005; Oberdorster et al. 1994).

The above-mentioned studies did not test exactly the same nanoparticles for the in vitro and in vivo set-ups. Arai et al. (2014) did test the same silver nanoparticles in vitro and in vivo. However, they did not measure the in vitro NP translocation but the presence of silver material in cells. They concluded that, in vivo, silver ions appear to translocate from the lungs to other tissues rapidly. In vitro, Ag from AgNO_3_ binds to metallothioneins, whereas Ag nanoparticles accumulate in lysosomes (Arai et al. 2014). Multi-walled carbon nanotubes (MWCNTs) were jointly tested in vitro and in vivo. However, this study focused on the development of pulmonary fibrosis and did not measure tissue distribution or translocation. The results did show, however, that MWCNTs are taken up by alveolar macrophages both in vivo and in vitro (Taylor et al. 2014).

## Intestinal barrier

Several in vivo oral studies have been performed, but only a very limited number of those in vivo studies can directly be compared to in vitro studies. Here, we focus only on studies that evaluated the uptake of food-relevant nanoparticles such as silica and titanium dioxide. In addition, studies that used model materials such as polystyrene are discussed, as polystyrene nanoparticles have also been used in vitro (Table 1).

Some studies investigated the in vitro and in vivo translocation of titanium dioxide across the gut epithelium. Six hours after a single oral administration of 130-nm TiO_2_, titanium could be detected in gut tissues, but due to the low concentrations, it could not be quantified (Brun et al. 2014). Janer et al. (2014) did not observe any increase in tissue concentration in vivo 24 h after a single dose of 100 mg/kg TiO_2_ of 18 nm, while very low translocation was observed in vitro (Janer et al. 2014). In another study, rats were exposed to 5 mg/kg of different types of TiO_2_ nanoparticles (mean particle size 40 nm, 40–50 nm, 120 nm and up to 5 µm), and up to 96 h post-administration, no translocation of titanium was detected to blood, several organs and urine. Also, no translocation was observed in vitro (MacNicoll et al. 2015). In rats exposed for 30 days to 200 mg/kg body weight (bw) 75-nm TiO_2_ nanoparticles, no increased titanium levels were detected in blood, liver kidney and spleen, while effects on liver and other organs have been reported (Wang et al. 2013). Titanium nanoparticles were also orally administered to rats, with a dose of 1 or 2 mg/kg TiO_2_ nanoparticles with a primary size of 20–60 nm (large agglomerates were present) for 5 days (Tassinari et al. 2014). Only a limited number of tissues were collected; interestingly, in spleen elevated Ti concentrations were found in the high dose group. Detailed analysis revealed the presence of 130-nm (sp ICP-MS) or 200–400-nm (SEM-EDX) materials in spleens (Tassinari et al. 2014).

Like titanium dioxide, silica is commonly used as food additives. Therefore, the oral uptake of silica nanoparticles was studied after 28 and 90 days of exposure to food-grade synthetic amorphous silica. Limited uptake was observed: only after 90-day exposure to 2500 mg/kg bw elevated Si levels were found in spleen (van der Zande et al. 2014). More studies are available that focussed on model polystyrene nanoparticles, and they generally highlight the dependence of uptake and accumulation of polystyrene nanoparticles on several factors, including their size, surface charge, and type of coating material (Araujo et al. 2014; Hillery et al. 1994; Hillyer and Albrecht 2001; Hussain and Florence 1998; Hussain et al. 1997; Jani et al. 1989). In general, smaller polystyrene nanoparticles were taken up across the GI tract to a higher extent than the larger ones (Jani et al. 1990); the non-ionic more than the carboxylated ones (Jani et al. 1989) and 407 poloxamer-coated more than 188 poloxamer-coated nanoparticles (Hillery and Florence 1996; Hussain et al. 1997).

The estimated oral bioavailability of 50-nm polystyrene nanoparticles varied between studies from 0.2 to 1.7 % (Walczak et al., submitted) to 6.6 % (Jani et al. 1990). Strikingly, much higher bioavailability (23 %) was reported for 500-nm polystyrene nanoparticles (Hussain et al. 1997), while their 1-µm-size counterpart’s nanoparticles had a lower uptake (2 × 10^−6^ % detected in lymph fluid) (Seifert et al. 1996). Also, the amounts of polystyrene nanoparticles associated with intestinal tissues that were reported by Walczak (between 0.38 and 0.74 % depending on the type of polystyrene nanoparticles, calculated as a sum of the small- and large intestinal walls), were lower than the ones reported by others for 60-nm nanoparticles (between 1.5 and 10 %, depending on the type (i.e. surface chemistry) of polystyrene nanoparticles used) (Hillery and Florence 1996; Hillery et al. 1994). Comparison of the oral in vivo bioavailability (0.2 and 1.7 %) (Walczak 2015), with the in vitro translocation values (1.6 to 12.3 %) of the same 50-nm polystyrene nanoparticles (Walczak et al. 2014), shows lower uptake values in the in vivo model. Therefore, the in vitro model used by Walczak et al. appears to overestimate the in vivo translocation.

## Skin barrier

In the literature, various in vitro assays (based on ex vivo skin tissue) have been used to determine percutaneous penetration of nanoparticles. However, the scarcity of in vivo human data hampers proper evaluation of these models. The vast majority of in vitro studies focused on TiO_2_ and ZnO nanoparticles found in sunscreens. Most, although not all in vitro studies find that these nanoparticles do not penetrate beyond the superficial layers of the stratum corneum (Cross et al. 2007; Mavon et al. 2007; Wu et al. 2009; Zvyagin et al. 2008). One of the disadvantages of in vitro assays is that exposure duration is limited to 24 h, although long-term exposures would be closer to the real-life situation. Wu et al. (2009) compared in vitro and in vivo penetration of nanoscale TiO_2_ (4 nm and 60 nm) in two animal models (hairless mice and pigs). After in vitro dermal exposure, TiO_2_ nanoparticles were not detected beyond the stratum corneum. However, in vivo, 30-day dermal exposure to the same nanoparticles in hairless mice revealed, that in contrast to short-term in vitro exposure, TiO_2_ nanoparticles do reach viable skin layers (Wu et al. 2009). Furthermore, after 60-day dermal exposure, TiO_2_ nanoparticles could penetrate through the skin, reach different tissues and induce diverse pathological lesions in several major organs (Wu et al. 2009). Deeper, although minimal, penetration into epidermal layers has also been found for ZnO nanoparticles in an in vivo study in human volunteers (Leite-Silva et al. 2013). In a parallel in vitro–in vivo study, Monteiro-Riviere et al. (2011) investigated the penetration of TiO_2_ and ZnO nanoparticles in UVB-damaged porcine skin. Under both conditions, TiO_2_ and ZnO NP predominantly resided in the stratum corneum, although small amounts of TiO_2_ and ZnO were also detected in the viable skin layers. On the other side, not all in vivo studies report penetration of nanoparticles beyond the stratum corneum (Monteiro-Riviere et al. 2011). For instance, Zvyagin et al. found in an in vivo study with human skin no penetration of ZnO (26–30 nm) into the viable layers (Zvyagin et al. 2008). This is consistent with the findings reported by Mavon et al. showing no penetration of TiO_2_ into the viable epidermal layers of human skin either in vivo or in vitro (Mavon et al. 2007). Obviously, the discrepancy in the results cannot be explained solely by the differences between in vitro and in vivo data but also by the characteristics of the used nanoparticles (size, coating), exposure duration and sensitivity of the detection methods.

Another frequently investigated NP is silver, which is used on a large scale in medicinal and consumer products. Larese et al. found in an in vitro study with human skin that smaller silver nanoparticles (30 nm) can penetrate across the stratum corneum into the upper layers of the epidermis (Larese et al. 2009). This is consistent with in vivo data obtained in human volunteers (George et al. 2014) showing that silver nanoparticles could penetrate as deep as the reticular dermis. Zhu et al. showed, using a highly sensitive detection method, that the penetration depth of Ag nanoparticles could exceed the stratum corneum thickness (Zhu et al. 2015).

## Placental barrier

As described above, the transport efficiency of nanoparticles across the human placenta is likely to be different from that in rodents (Wick et al. 2010). To study the translocation over the human placental barrier, dual recirculation human placental (ex vivo) perfusion models are used. In such a design the translocation of 50, 80-, 240-, or 500-nm polystyrene was studied, under highly controlled conditions (i.e. translocation of a marker compound) (Wick et al. 2010). After a single administration, 50-, 80-, and 240-nm polystyrene nanoparticles were observed in the foetal circulation (foetal to maternal ratios 0.4; 0.4; 0.1, respectively), while the 500-nm polystyrene nanoparticles were retained in the placenta (Wick et al. 2010). In vitro similar results were found, where the translocation rate of 50-nm polystyrene nanoparticles was larger compared with 100-nm nanoparticles (Cartwright et al., 2012). Myllynen et al. perfused ex vivo human placentas with 10-, 15-, and 30-nm PEGylated gold nanoparticles (for up to 6 h) and did not observe any PEGylated gold nanoparticles in the foetal part of the placenta (Myllynen et al. 2008). In vitro, they observed internalization of PEGylated gold nanoparticles of 10 nm in BeWo cells up to 48 h after exposure (Myllynen et al. 2008).

In rodents, fluorescent polystyrene nanoparticles were administered via the extraembryonic tissue. The embryos were ex vivo incubated for 12 h with 20-, 100-, and 500-nm carboxylated and 200-nm amino-conjugated/terminated polystyrene nanoparticles. The 20-nm carboxylated nanoparticles were distributed in the embryonic and extraembryonic germ layers of ectoderm, mesoderm, and endoderm. The 100 and 500-nm carboxylated polystyrene nanoparticles accumulated in extraembryonic tissue. Interestingly, the 200-nm amino-conjugated particles can pass into the embryos (Tian et al. 2009). For 5-nm gold nanoparticles, 0.018 % of the administered dose (118 µg/kg bw intravenous) administered to rats at day 19 of gestation was detectable in the embryo (Takahashi and Matsuoka 1981).

In addition, studies with silica nanoparticles showed that 70 nm, but not 300 or 1000 nm reached the brain and liver of the foetus after i.v. administration to the mother (Yamashita et al. 2011), while there was limited transport of 25-nm silica nanoparticles in vitro (Sonnegaard Poulsen et al. 2013). In the same study of Yamashita et al. 63-nm TiO_2_ nanoparticles were detected by TEM in brains and livers of foetuses (Yamashita et al. 2011). In another study, surface modified 28–30-nm iron oxide nanoparticles were administered (intraperitoneal) to mice from gestation days 9–16. Nanoparticles with a positive zeta potential in water [coated with hydrophilic polyethyleneimine (PEI)] were detected in the livers of foetuses 1 day after dosing of the dams, while negatively charged nanoparticles (coated with acrylic acid) could not be found in the foetuses (Di Bona et al. 2014). In vitro, iron oxide nanoparticles were able to cross a BeWo cell layer (Correia Carreira et al. 2013).

Based on these studies, it can be concluded that nanoparticles can pass the placenta of rodents and humans (ex vivo). This translocation is size- and surface charge-dependent. Although there are few studies available to compare the in vitro translocation to the in vivo or ex vivo translocation, for polystyrene nanoparticles, size-dependent translocation has been observed both ex vivo and in vitro. However, for gold nanoparticles and iron oxide nanoparticles the in vitro translocation was higher compared with the ex vivo or in vivo translocation, while for silica nanoparticles it was lower.

## Analytical techniques to quantify and characterize nanoparticle translocation in vitro and in vivo

Meaningful interpretation and comparison of the results obtained using different in vitro experiments and extrapolation to in vivo data require reliable characterization of the nanoparticles and their aggregates, as well as matrix-based influences on the nanoparticles. Therefore, appropriate analytical techniques should be applied to determine the nanoparticle size distribution, composition, and concentration in the experimental samples.

Widely used methods to detect nanoparticles in liquid dispersions are dynamic light scattering (DLS), centrifugal liquid sedimentation (CLS) (Braun et al. 2011; Cascio et al. 2015; Murdock et al. 2008; Nickel et al. 2014; Powers et al. 2006) and nanoparticle tracking analysis (NTA) (Filipe et al. 2010; Vasco et al. 2010). These methods allow determining an average size or size distribution related to the measured intensity signal. NTA is able to count and size nanoparticles in aqueous media at µg/L to mg/L concentrations (Filipe et al. 2010; Vasco et al. 2010). Both DLS and NTA are highly dependent on the polydispersity of the nanoparticle suspension and material properties of the particles since the scattered light of the individual particles must be sufficiently strong for detection. CLS is more robust since particles are size-separated before their actual detection and sizing. Transmission and scanning electron microscopy (TEM, SEM) are techniques to visualize nanoparticles (Dudkiewicz et al. 2011; Zhang et al. 2012). If pure nanoparticle dispersions are analysed, EM is currently the only technique that reliably covers the entire size range down to 1 nm. In cells or tissues, the minimal particle size that can be detected is around 20 nm, depending on the electron density of the nanoparticles (De Jong et al. 2010). Furthermore, EM distinguishes size aggregates and primary particles. Other imaging techniques are atomic force microscopy (AFM)(Brown et al. 2013) and particle-induced X-ray emission (PIXE) spectroscopy (Lozano et al. 2012, 2013).

Elemental information about the sample can also be obtained by atomic spectrometry methods such as inductively coupled plasma optical emission spectrometry (ICP-OES) (Elzey et al. 2012) and ICP mass spectrometry (ICP-MS) (Krystek 2012; Krystek et al. 2013), especially in single particle mode (sp ICP-MS) (Laborda et al. 2014; Pace et al. 2011; Peters et al. 2014a). From these studies it becomes clear that he smallest particle sizes that now can be determined are around 20 nm for silver and gold nanoparticles. For TiO_2_ and SiO_2_, nanoparticles size detection limits are around 50 and 200 nm; however, recent experiments suggest that the size detection limit may become lower in the next few years. Another promising possibility is to combine size-specific techniques, that separate particles from each other, such as hydrodynamic chromatography (HDC) or field-flow fractionation (FFF) with atomic spectroscopy techniques, that characterize particles, such as ICP-MS (Bednar et al. 2013; Hassellov et al. 2008; Peters et al. 2014b; Striegel and Brewer 2012; Von der Kammer et al. 2011). Currently, asymmetric flow field-flow fractionation (AF4) is the most successfully used variant of FFF (Zattoni et al. 2014). In in vitro testing, AF4 coupled to ICP-MS becomes a powerful tool to investigate, for example, time-dependent uptake of medium-sized silver nanoparticles (Krystek et al. 2015). Laser ablation (LA)-ICP-MS has been used to quantify gold nanoparticles in single cells (Wang et al. 2014).

Most of the detection methods described above generally require sample preparation procedures. However, only little information concerning sample preparation techniques is available in the literature (Szakal et al. 2014). Aqueous media containing nanoparticles only need limited sample preparation; samples may be sonicated or tip-sonicated to suspend materials and proteins such as bovine serum albumin (BSA), or detergents such as sodium dodecylsulphate (SDS) may be added to stabilize nanoparticle suspensions (Jensen et al. 2011). For other matrices, matrix removal or nanoparticle isolation from the matrix can be achieved by physical processes such as centrifugation, filtration, column techniques or cloud point extraction, or by chemical or enzymatic destruction of the matrix (Loeschner et al. 2014; Peters et al. 2014a, b).

As mentioned not only size, but also, surface chemistry and charge, and composition of the so-called protein corona have been shown to significantly affect the translocation of nanoparticles. The nanoparticle surface charge (or zeta potential) is routinely determined, but a correct interpretation might be hampered by interactions of the matrix with these measurements. More details analysis of the surface of the nanoparticles, for example, by matrix-assisted laser desorption/ionization time-of-flight mass spectrometry (MALDI-TOF) should be considered (Walczak et al. 2014). Methods for the assessment of protein composition range from simple gel electrophoreses experiments to a full characterization of the biomolecules that are present using mass spectrometry-based techniques (Lesniak et al. 2010; Lundqvist et al. 2008; Tedja et al. 2012).

Overall, different techniques are available to determine the nanoparticle size distribution, composition and concentration in experimental samples. Attention should be paid to the limits of the chosen techniques and to adequate sample preparation; this should be incorporated in studies reporting translocation data.

## Conclusions on comparison of in vitro translocation models with in vivo data

While comparing the in vitro translocation to in vivo data, we noticed that very few studies tested exactly the same nanoparticles in in vitro and in in vivo settings. It seems that most studies are performed either in vitro or in vivo. Therefore, we focused our comparison on the nanoparticle chemical composition although the nanoparticles studied sometimes differ in other particle characteristics such as size. Interestingly, this approach revealed that for some nanoparticles, the in vitro translocation is similar to the in vivo translocation. Examples are the translocation of polystyrene and gold nanoparticles in in vitro lung barrier models, and the translocation of titanium dioxide and silver nanoparticles in in vitro skin models. However, for other nanoparticles, major differences seem to exist between the in vitro translocation rate and the in vivo translocation.

These differences in translocation may be caused by the many differences existing between the in vitro barrier models and the in vivo study designs. First of all, the in vitro models mostly use an acute exposure of maximum 24 h and a short post-exposure time, while, for example, in vivo inhalation studies vary from 1 h to 13 weeks of inhalation exposure, 5 days a week, 6 h per day with post-exposure periods of up to 1 year. The relative short in vitro exposure durations might imply that only a fraction the nanoparticles that have been taken up (intracellularly) has been translocated. Long-term exposures are not conceivable in an in vitro set-up, although this would be a more realistic exposure scenario. Second, exposure concentrations between the in vitro and in vivo studies differ. In in vitro experiments, high particle concentrations are sometimes used to be able to detect the nanoparticles in the basolateral compartment. These high concentrations might increase the agglomeration state of the nanoparticles and damage the epithelial barriers resulting in a different translocation mechanism compared with the in vivo situation. Clearly also in vivo dose selection can be critical, as it has been shown in rats that exposure to high concentrations to silica nanoparticles cause gelation (agglomeration) of silica nanoparticles in the gut (van der Zande et al. 2014). Third, in vitro models have a single basolateral compartment, while in vivo, nanoparticles can translocate to various tissues and organs. In the in vitro set-up, saturation might occur while under in vivo conditions nanoparticles can continue translocating, since they are taken up in organs and removed from the blood. On the other hand, this distribution of nanoparticles to various tissues can hamper translocation studies in vivo. Fourth, current in vitro models are almost all static, while in vivo exposure is dynamic. Fifth, nanoparticles adsorb proteins and/or phospholipids in biological fluids such as serum or lung-lining fluid (Landsiedel et al. 2014b). These proteins or phospholipids form a corona around the particles that affects their uptake and bioavailability (Lesniak et al. 2012). The proteins that are encountered in in vitro models, for example in foetal calf serum, are completely different from the proteins in vivo (i.e. rodent or human). Sixth, many in vitro lung models are submerged, which might alter the nanoparticle characteristics and thus the translocation rate, while most in vivo studies rely on inhalation exposure.

## Conclusions

The aim of this review was to evaluate the performance of in vitro models that mimic different physiological barriers found in the human body by comparing—when possible—the in vitro translocation of nanoparticles to their in vivo translocation across the lung, gut, skin, and placental barriers. For all these barriers, a great variety of in vitro models are available to evaluate the translocation of nanoparticles, ranging in complexity from single-cell-type monolayer to multi-cell (3D) models. Many studies that use in vitro models on inserts focus on the toxicity of nanoparticles, do not include their translocation, and were thus not included in this review. Clearly, for a correct interpretation of the observed toxicity, the translocation (or systemic availability, internal concentration) is a crucial parameter. In recent years, the availability of analytical detection methods to quantify and characterize the nanoparticles in in vitro settings has improved considerably, which provides high-quality data that are valuable in studying the relationship between physiochemical properties of the nanoparticles and their translocation. The improved analytical chemical detection methods also contributed to an increase in in vivo uptake data (bioavailability) of nanoparticles.

Here, while comparing the in vitro translocation to in vivo data, we noticed that very few studies tested exactly the same materials in both settings. Comparing data obtained using nanoparticles of the same chemical composition, we found that for some nanoparticles, the in vitro translocation is similar to the in vivo bioavailability. Examples are the translocation of polystyrene and gold nanoparticles in in vitro lung barrier models, and that of titanium dioxide and silver nanoparticles in in vitro skin models that are both in line with the in vivo data. However, for other nanoparticles, major differences were found between the in vitro and the in vivo translocation rate. As discussed in the sections above, many differences exist in the experimental set-up between in vitro and in vivo study design that probably account for the poor correlation between these two types of studies. Especially, the changes in the physicochemical characteristics of the nanoparticles caused by the presence of lung-lining fluid, mucus, serum protein, and lipoproteins that form a corona should be taken into account, as these dramatically alter their recognition, uptake and translocation (Lesniak et al. 2012; Treuel et al. 2013).

Risk assessment of nanoparticles (as is true for chemicals in general) still heavily relies on in vivo studies using experimental animals. However, the latter must be reduced as far as possible, for numerous reasons. Therefore, there is an urgent need to validate existing in vitro models using data from animal models, although these animal models do not fully simulate the physiology of humans. How can we establish an in vitro barrier model that has value for the risk assessment of nanoparticles for humans? Ideally, in vitro models reflect the key mechanisms of corresponding in vivo end points, which cannot always be accomplished and may not be required if in vitro models reliably detect nanoparticles that are of concern in vivo (Landsiedel et al. 2014b). The predictive value of in vitro models can be better assessed by testing exactly the same nanoparticles simultaneously in both in vitro and in vivo assays. If the ranking of the tested nanoparticles from the lowest translocation rate to the highest translocation rate is the same in both cases, the in vitro models provide information on the internal exposure, which is critical for the ultimate systemic adverse effects. Then, the in vitro models can be considered as suitable for the risk assessment of nanoparticles and will in addition help to reduce animal testing by setting priorities for subsequent in vivo testing.

A crucial factor for both translocation studies is the sensitivity of the analytical technique used for the quantification of the translocation. While this is of importance also for in vivo studies, in vitro studies are more vulnerable for poorly performing methods. The observed low translocation rates combined with the relatively short exposure durations in vitro pose great analytical challenges. The absence of acute in vitro translocation should always be interpreted in relation to the sensitivity of the detection technique (both in terms of concentration and nanoparticle size). The introduction of in vitro models that allow chronic exposures are promising in that a chronic exposure better reflects the real-life human exposure.

In vitro models with a high predictive value do not necessarily have to be complex, but can consist of a single cell type, as long as they give a similar ranking of nanoparticles as obtained in the in vivo situation. However, the mechanism in these models might be completely different from the in vivo situation. To obtain more insight into the mechanisms behind nanoparticle uptake and translocation, in vitro models should be further developed to become physiologically very close to the in vivo human conditions. Such in vitro models must be more complex because the human lung, gut, skin, and placental barrier consist of multiple cell types, which are exposed to a low concentration of nanoparticles. A physiologically realistic model will increase the confidence in the NP testing outcome, but it will also be elaborate and expensive to develop and maintain.

When in vitro models are available that either have a high human predictive value or are physiologically similar to the human situation, experimental data can be used to develop in silico models that will eventually be able to predict the human in vivo bioavailability of nanoparticles from their in vitro translocation rate. The (improved) in vitro models still needs validation, most likely using animal data, clearly alternative approaches need to be developed for this (making use of available human data).

We conclude that the current in vitro models to study the translocation of nanoparticles do not (yet) allow correlating to the reported in vivo translocation because of many differences between the in vitro and in vivo study designs. However, the use of in vitro models is very promising since they are currently further improved to mimic the in vivo situation more closely by, for example, using co-cultures of different cell types and implementing them in a microfluidic format. When these models are further validated by testing exactly the same nanoparticles in an in vivo set-up as in the in vitro model, then, they can be used to determine the internal exposure (bioavailability) of nanoparticles and to set priorities for nanoparticles testing.
